# Preclinical characterization of the JAK/STAT inhibitor SGI-1252 on skeletal muscle function, morphology, and satellite cell content

**DOI:** 10.1371/journal.pone.0198611

**Published:** 2018-06-13

**Authors:** Jacob R. Sorensen, Jordan D. Fuqua, Michael R. Deyhle, Jacob Parmley, Caitlin Skousen, Chad Hancock, Allen C. Parcell, Robert D. Hyldahl

**Affiliations:** 1 Department of Exercise Sciences, Brigham Young University, Provo, Utah, United States of America; 2 Department of Nutrition, Dietetics and Food Science, Brigham Young University, Provo, Utah, United States of America; University of Minnesota Medical Center, UNITED STATES

## Abstract

**Background:**

Recent studies have highlighted the JAK/STAT signaling pathway in the regulation of muscle satellite cell behavior. Herein we report preclinical studies designed to characterize the effects of a novel JAK/STAT inhibitor on plantar flexor skeletal muscle function, morphology, and satellite cell content.

**Methods:**

The compound, SGI-1252, was administered orally (400mg/kg) in a 10% dextrose solution to wild type mice (n = 6) 3 times per week for 8 weeks. A control group (n = 6) received only the dextrose solution.

**Results:**

SGI-1252 was well tolerated, as animals displayed similar weight gain over the 8-week treatment period. Following treatment, fatigue in the gastrocnemius-soleus-plantaris complex was greater in the SGI-1252 mice during a 300 second tetanic contraction bout (p = 0.035), though both the rate of fatigue and maximal force production were similar. SGI-1252 treated mice had increased type II myofiber cross-sectional area (1434.8 ± 225.4 vs 1754.7 ± 138.5 μm^2^), along with an increase in wet muscle mass (125.45 ± 5.46 vs 139.6 ± 12.34 mg, p = 0.032) of the gastrocnemius relative to vehicle treated mice. SGI-1252 treatment reduced gastrocnemius STAT3 phosphorylation 53% (94.79 ± 45.9 vs 44.5 ± 6.1 MFI) and significantly increased the concentration of Pax7^+^ satellite cells (2589.2 ± 105.5 vs 2859.4 ± 177.5 SC/mm^3^) in the gastrocnemius. SGI-1252 treatment suppressed MyoD (p = 0.013) and Myogenin (p<0.0001) expression in human primary myoblasts, resulting in reduced myogenic differentiation (p = 0.039).

**Conclusions:**

Orally delivered SGI-1252 was well tolerated, attenuates skeletal muscle STAT3 activity, and increases satellite cell content in mouse gastrocnemius muscle, likely by inhibiting myogenic progression.

## Introduction

Skeletal muscle regeneration and the maintenance of muscle mass are attributed, in large part, to the activity of muscle satellite cells [1, 2]. Under normal conditions, satellite cells exist in a quiescent state, expressing the satellite cell marker Pax7. When activated by injury or exercise, satellite cells undergo multiple rounds of proliferation. A portion of the cells terminally differentiate and fuse to the existing myofibers to support regeneration, maintenance, and/or growth of the muscle fiber [3, 4]. The cells that do not commit to differentiation return to quiescence and serve to maintain the satellite cell pool [5, 6]. The ability of satellite cells to cycle between self-renewal and quiescence is critical in preserving skeletal muscle function [7–10]. In muscle wasting conditions, such as aging, satellite cell quiescence and self-renewal characteristics are altered, resulting in decreased muscle mass, strength, and function [11, 12]. Satellite cell dysfunction is linked with factors that affect the muscle niche, such as increased adipose and fibrotic tissue accumulation in conjunction with chronic inflammation [13, 14]. However, it has been demonstrated that an intrinsic shift in satellite cell signaling, indicative of cellular stress [15, 16], or potentially caused by the cellular environment [7, 10], can also contribute to a dysfunctional and depleted satellite cell pool. Inhibition of the Janus Kinase/Signaling Transducer and Activator of Transcription (JAK/STAT) pathway has been identified as a potential therapeutic target for attenuating satellite cell dysfunction [17, 18].

Stress to skeletal muscle initiates the release of cytokines, hormones, and growth factors that activate the JAK/STAT pathway [19]. JAK is a family of tyrosine kinases that auto-phosphorylate and attract cytoplasmic binding of STAT proteins. The STAT family proteins can stimulate multiple pathways or dimerize and translocate to the nucleus, induce mRNA transcription, and promote synthesis of myogenic proteins [20]. Specifically, STAT3 activation is crucial in embryonic development [21] and is known to regulate myogenic progression of adult satellite cells [22, 23]. Marked increases in basal levels of STAT3 have been identified in muscle wasting conditions in humans [24] and mice [18, 25]. Notably, in these situations, satellite cells lose the characteristic of reversible quiescence [10], compromising the self-renewing process, and diminishing the satellite cell pool [26]. However, *in vitro* inhibition of STAT3 activity prompts an expansion in the satellite cell pool by attenuating differentiation of the dividing cells [17]. In mice, pharmacological STAT3 inhibition also enhanced recovery time following injury, attributed to the preservation of satellite cell quiescence [18]. Combined, these results indicate that STAT3 inhibition via a pharmacological intervention, may be advantageous in treating conditions that result in satellite cell dysfunction by protecting satellite cell quiescence and self-renewal characteristics.

As JAK/STAT signaling is known to play a significant role in the pathogenesis of myeloproliferative disorders and rheumatoid arthritis, the development of JAK/STAT inhibitors have recently received considerable attention as a pharmacologic intervention for these conditions [27, 28]. Along this same vein, Ahmed *et al*. had previously developed a small molecule (SGI-1252) which showed high antagonist activity against both JAK1 (IC_50_ = 14.8nM) and JAK2 (IC_50_ = 2.0) [29]. They further showed that SGI-1252 was an orally bioavailable molecule that was well-tolerated in mice [29]. Therefore, the aim of the present study was to determine its feasibility as a potential therapeutic intervention for skeletal muscle disorders characterized by satellite cell dysfunction. Specifically, we investigated the effects of orally administered SGI-1252 on plantar flexor skeletal muscle function, morphology and satellite cell content in healthy young mice. We hypothesized that SGI-1252 would effectively decrease skeletal muscle STAT3 activity, and result in an expansion of the satellite cell pool without detrimental effects to muscle size and function.

## Methods

### Animals

Twelve C57BL/6J mice, 4–6 weeks of age, were randomly assigned into a treated (n = 6) or vehicle control group (n = 6). These mice were part of a larger study that required that the mice be of the designated age. The mice were kept on a 12-hour light-dark cycle (6am to 6pm) and were fed standard chow and water ad libitum. All procedures and methods were approved by the Institutional Animal Care and Use Committee (Reference Number: 140304). All surgery was performed under isoflurane anesthesia, and all efforts were made to minimize suffering.

### Research design

Each group was treated three times per week (Monday, Wednesday, and Friday) for 8 weeks using a modified gavage technique of either 400 mg • kg^-1^ body weight of the SGI-1252 solution (160 mg• ml^-1^ in 10% dextrose) or vehicle (10% dextrose). The modified technique was chosen to more closely replicate human administration of the drug and to limit any additional stress placed on the mice. During gavage, the mice were secured by scruffing the back of the neck, and the solution was delivered by inserting a pipet tip (1000 μl) to the back of the throat while slowly ejecting the liquid. Scruffing was maintained until the bolus of liquid was no longer visible and the mice swallowed.

### Functional measures

For functional and histological measures, we chose to focus our investigations on the plantar flexor muscle group, as these muscles have been shown to preferentially lose satellite cell content over the lifespan relative to other lower limb muscles [30]. Following 8 weeks of treatment, isometric tetanic contractions of the gastrocnemius-plantaris-soleus (GPS) muscle complex were measured using an Aurora Scientific 1500A muscle test system while mice were fully anesthetized. The skin of the lower limb was removed to expose the GPS complex and the distal tendon was cut and secured to the force transducer. The mouse was placed on a heated surgical plate and the femur was secured between two pins to isolate the GPS force. The sciatic nerve was exposed and tied off with thread to allow for direct electrode-to-nerve stimulation to elicit contractions. Maximal isometric tetanic force production, sustained force, and force loss were determined from sciatic nerve stimulation every 2 seconds for 300 seconds with a Grass S88X stimulator. The stimulation parameters were 100ms train duration consisting of 15, 0.05-ms square wave pulses (150 Hz) with 3–5 V [31, 32]. After completing the functional measurements, animals were immediately euthanized via exsanguination and cervical dislocation and muscles were harvested, cleaned and weighed. The stimulated muscle was mounted on a section of cork with Tragacanth gum and frozen in liquid nitrogen-cooled isopentane for immunohistochemical analysis. Non-stimulated muscle samples were flash frozen in liquid nitrogen for protein analysis. Muscle samples were stored at -80˚C.

### Histology

Frozen tissue samples were serially sectioned at 8 μm using a cryostat microtome (Microm HM 525, Thermo Scientific) and mounted on Superfrost slides. For assessment of myofiber cross sectional area and satellite cell content, tissue cross sections were immunostained for type I myosin heavy chain (MyHC), dystrophin or Pax7. Sections were air-dried for 10 min. and then fixed in 2% paraformaldehyde (Sigma-Aldrich) for 8–10 min. Following fixation of slides that were stained for MyHC, sections were washed in phosphate-buffered saline (PBS) solution 3 times for 2 min. Sections were then blocked in 0.2% Triton X-100, 2% bovine serum albumin (BSA), 5% fetal bovine serum (FBS) solution for 30 min at room temperature in a humidified container. Following fixation of slides that were stained for Pax7, sections were washed in PBS 2 times for 5 min. Sections were then blocked using M.O.M (Vector Laboratories) blocking reagent for 90 min, washed, and incubated in M.O.M. diluent (Vector Laboratories) for 5 min. Sections for both stains were incubated in the appropriate primary antibody in a humidified chamber overnight at 4°C. Following several washes, sections were then incubated in the appropriate secondary antibody for 30 min at 37°C. Sections were then washed multiple times and rinsed in ultra-pure water. Stained slides were then dried and mounted using Fluoroshield histology mounting medium (Sigma–Aldrich). The following primary antibodies were used: myosin heavy chain I, IgG2b (1:100; Developmental Studies Hybridoma Bank; BA-D5), dystrophin lgG (1:200; abcam), Pax7, IgG1 (concentrate 1:50; Developmental Studies Hybridoma Bank). Secondary antibodies used were: Rhodamine Red IgG2b (1:100; Jackson ImmunoResearch Laboratories), Cy3 lgG (1:100; Jackson ImmunoResearch Laboratories), Alexa Fluor 488 IgG1 specific (1:100; Jackson ImmunoResearch Laboratories), DAPI (1:150; Thermo Scientific).

Analyses of immunofluorescent images were carried out by an investigator that was blind to treatment group. The entire section was imaged using a 20× objective (~9 images). All images were quantified and satellite cell numbers were expressed as the number of Pax7^+^ cells per volume (mm^3^) of muscle tissue [33]. Quantification of myofiber type, cross-sectional area measurements were made using 4×, 10× and 20× images, respectively of the sample for type I myosin heavy chain-stained sections [34]. Myofibers were traced manually and quantified using Olympus cellSens software. An average of 37.4 ± 16.6 type I fibers and 124 ± 15 type II fibers were analyzed per animal. Fiber circularity was calculated as (4π•CSA)/(perimeter)^2^, all fibers below 0.60 were excluded from analysis [35]. No differences in fiber circularity were observed between groups (*p* = 0.73), validating the comparisons for muscle fiber CSA. Type I fibers are relatively low and localized to the medial head of the gastrocnemius in C57BL6J mice [36] such that the total number of type I fibers was counted in each animal. Fiber type distribution was performed by randomly selecting an image from the medial head location and both type I and II fibers were counted and calculated as a percentage of the total number of fibers. Myonuclei were assessed using 40× images. Nuclei were considered myonuclei when more than 50% of the cell mass was located within the dystrophin perimeter [37].

### Protein analysis

Frozen tissue samples were weighed (133.2 ± 11.7 mg) and homogenized in lysis buffer (cat# 43–040 from Millipore) at a ratio of 9 μL per mg tissue. HaltTM Protease and Phosphatase Inhibitor Cocktail (100X) (cat# 78440 from Thermo Fischer Scientific) was also added to the homogenate. Tissue disruption was performed on ice using a glass on glass dounce homogenizer. The homogenate was centrifuged at 10,000 g, 4° C for 10 minutes. The supernatant was analyzed in triplicate for total protein concentrations with the BCA Protein Assay Kit (product# 23227, Thermo Scientific Pierce). Supernatant was stored at -80° C. Total STAT and phosphorylated STAT3 protein concentrations were determined using a MAGPIX multiplexing platform (Luminex xMAP Technology). Analysis was performed using reagents supplied by Millipore according to the manufacturer's recommendations. In summary, antibody-conjugated magnetic beads were incubated with 25 μL of tissue homogenate (15 μg protein/well) overnight at 4° C on a plate shaker. Bead-complexes were then washed and incubated in detection antibodies for 1 hour on a plate shaker at room temperature. This was followed by incubation in streptavidin-conjugated phycoerythrin for 15 minutes on a plate shaker at room temperature, with an additional 15-minute incubation in amplification buffer. Bead-complexes were re-suspended in Assay Buffer and mixed on a plate shaker for 5 minutes, and then analyzed on a MAGPIX multiplex platform. Mean Fluorescent values were recorded and used for data analysis. Data was analyzed using Milliplex Analyst 5.1 software (Millipore Corporation, Billerica, MA).

### Biopsy and cell isolation

Human skeletal muscle biopsies and cell isolation were in accordance to previously published work [38] and approved by the Brigham Young University institutional review board (Reference Number: 14529). After signing a written informed consent document, muscle biopsies from the vastus lateralis of a healthy 32-year old male subject were weighed, cut, and digested enzymatically for 60 minutes at 37˚ C with titration every 15 minutes, filtered, and re-suspended in growth medium. Cells were incubated at 37˚ C with 5% CO_2_ for 7 days while changing the growth medium every 48 hours. After incubation, myoblasts were isolated using Immunomagnetic Bead Sorting based on CD56 expression [38]. This method has been shown to yield >95% myogenic cell purity [38]. Nevertheless, it is possible that contamination of other cell types is possible.

### Cell culture

Myoblasts were re-suspended in Dulbecco’s modified eagle medium (DMEM) supplemented with 20% Fetal bovine serum (FBS) with 1% penicillin and streptomycin plated at ~4,000 cells per cm^2^ on 24 well plates. Cultures were maintained at 37°C in a 5% CO_2_ incubator to proliferate until 60–70% confluent with the growth medium being changed every 48 hours. Cells were passaged and plated on collagen treated 24-well plates at a density of ~4,000 cells per cm^2^ for analysis. Cell culture experiments were performed on 3 separate occasions using 4 wells per treatment and the data were pooled. A portion of the cells were treated at a single time point with either a 100nM (n = 4) or 500nM (n = 4) SGI-1252 solution, or as a vehicle control in only the growth medium (n = 4), and 5-ethynyl-2’-deoxyuridine (EdU) used to assess proliferation at 6, 24, 48, and 72 hours post-treatment in accordance with Click-iT® EdU Alexa Fluor® 488 Imaging Kit protocols (ThermoFisher Scientific). The treatment concentrations were based on prior empirical testing and represent the highest concentrations that maintained normal cell viability and proliferation rates. 4',6-Diamidino-2-phenylindole dihydrochloride (DAPI) was used to measure total nuclei numbers. For differentiation experiments, growth medium was replaced with a differentiation medium consisting of 2% horse serum and 1% penicillin and streptomycin in DMEM with or without SGI-1252. Two doses of SGI-1252, 100nM (n = 8) or 500nM (n = 8) were used, while the control group (n = 8) received only the differentiation medium. To assess the expression of the myogenic regulatory factors during the early stages of differentiation, we cultured the cells in differentiation meduim for 3 days without changing the medium or re-treating the cells during that period of time. For the longer-term differentiation experiments (6 and 12 days), the cells were allowed to differentiate, and medium with the corresponding SGI-1252 or vehicle treatment was changed every 48 hours.

### Immunohistochemistry

At 24 and 72 hours, cultures were stained for nuclei, MyoD and Myogenin. At 6 days, cultures were stained for Myosin Heavy Chain (MyHC) expression. After incubation, cells were rinsed in phosphate buffered saline (PBS) and fixed in 3% paraformaldehyde (PFA) for 2 minutes. Following fixation, cells were permeabilized in 0.5% Triton® X-100 for 3 minutes followed by another 2 minutes of fixation in 3% PFA. Cells were rinsed twice in phosphate buffered saline with 20% tween (PBST) and then blocked in 3% bovine serum albumin (BSA) and 5% FBS for 30 min at room temperature. Cells were rinsed in PBS and incubated in the dark at room temperature in their respective primary antibodies (diluted in PBS at 1:100) for 60 minutes. Primary antibodies: MyoD—Monoclonal Mouse Anti-MyoD1 (Dako Company), Myogenin—Rabbit Polyclonal IgG Myogenin sc-576, Lot# G0313 (Santa Cruz Biotechnology), and Myosin Heavy Chain—MF 20-S mouse anti-myosin (Developmental Studies Hybridoma Bank). Following 3 rinses in PBST, cells were then incubated with 4',6-Diamidino-2-phenylindole dihydrochloride (DAPI) to stain nuclei and the appropriate secondary antibodies (diluted at 1:100 in PBS) for 60 minutes at room temperature in the dark. Secondary antibodies: MyoD—Alexa Fluor® 488-conjugated AffiniPure Goat Anti-Mouse IgG (H+L) (Life Technologies), Myogenin—Cy™3-conjugated AffiniPure Goat Anti-Rabbit IgG (H+L) (Jackson ImmunoResearch), and Myosin Heavy Chain—Alexa Fluor® 488-conjugated AffiniPure Goat Anti-Mouse IgG (H+L) (Life Technologies).

### Imaging and analysis

5 random sample images were collected from each well by investigators that were blind to both condition and time point using an Olympus IX73 microscope and Olympus XM10 camera. Proliferation time points at 6, 24, 48, and 72 hours were used to compare total nuclei to EdU^+^ nuclei. Differentiation analysis was done at 24 and 72 hours, assessing MyoD^+^ and Myogenin^+^ nuclei to total nuclei. For the 6 day analysis, total myotube area was measured and MyHC^+^ nuclei were compared to total nuclei. Images were analyzed using Olympus cellSens™ microscope imaging software. Additionally, time lapse images were collected every 15 minutes for 12 days using the Lonza CytoSMART™System (Lonza) to analyze HPM differentiation under the 500nM SGI-1252 and vehicle control conditions. HPM plate coverage over the 12-day culture period was measured by the CytoSMART software.

### Statistical analysis

Statistical analysis was performed on JMP Software version 12. A mixed model linear regression was performed to determine any differences in total body mass during the 8 week treatment protocol and in the analysis of fatigue for the 300 second tetanic contraction bout. All other analyses for the animal model were performed using an unpaired student t-test. Both two-way and one-way analysis of variance (ANOVA) was used to detect potential differences in cell culture experiments for the three treatment groups (500nM, 100nM or vehicle control). Significant interactions and main effects were further analyzed using the Tukey’s HSD post hoc test. All results are presented as means ± SD. Significance was accepted at p < 0.05.

## Results

### Animal weight and drug tolerability

**Fig 1:**
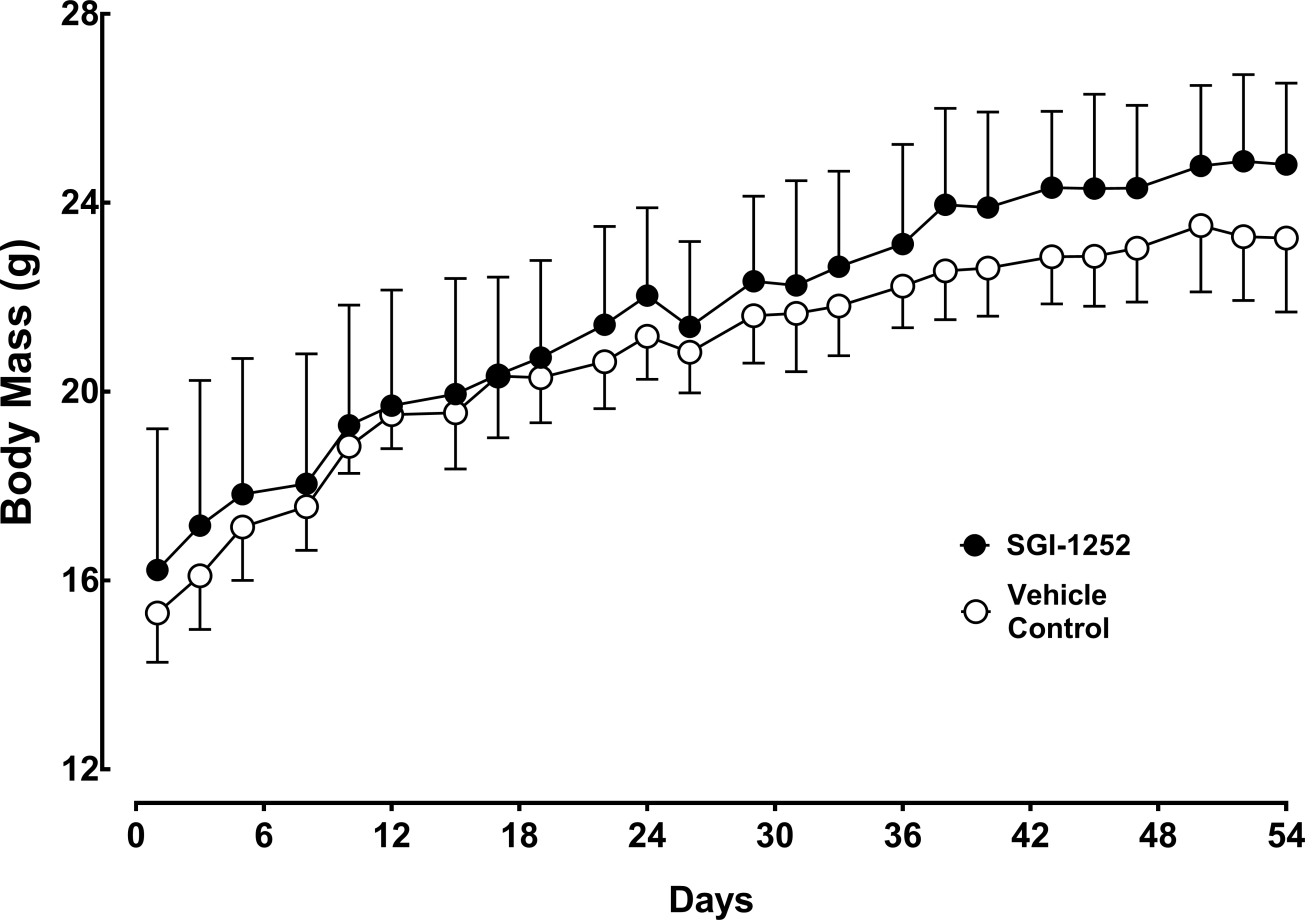
Body mass. Wild-type mice were weighed and treated 3 days a week for 8 weeks with either SGI-1252 or a vehicle solution. Data are means ± SD.

Twelve C57BL/6J mice, 4–6 weeks of age, were randomly assigned into a treated (n = 6) or vehicle control group (n = 6) and administered SGI-1252 three times per week over an eight-week period. Body weight increased similarly between the control and SGI-1252 treated mice through the 8-week treatment period (p = 0.8814, R^2^ = .972) (Fig 1). Furthermore, no significant adverse effects were noted in the treated group, indicating that the drug was well tolerated at the selected oral dose.

### Muscle functional characteristics

**Fig 2:**
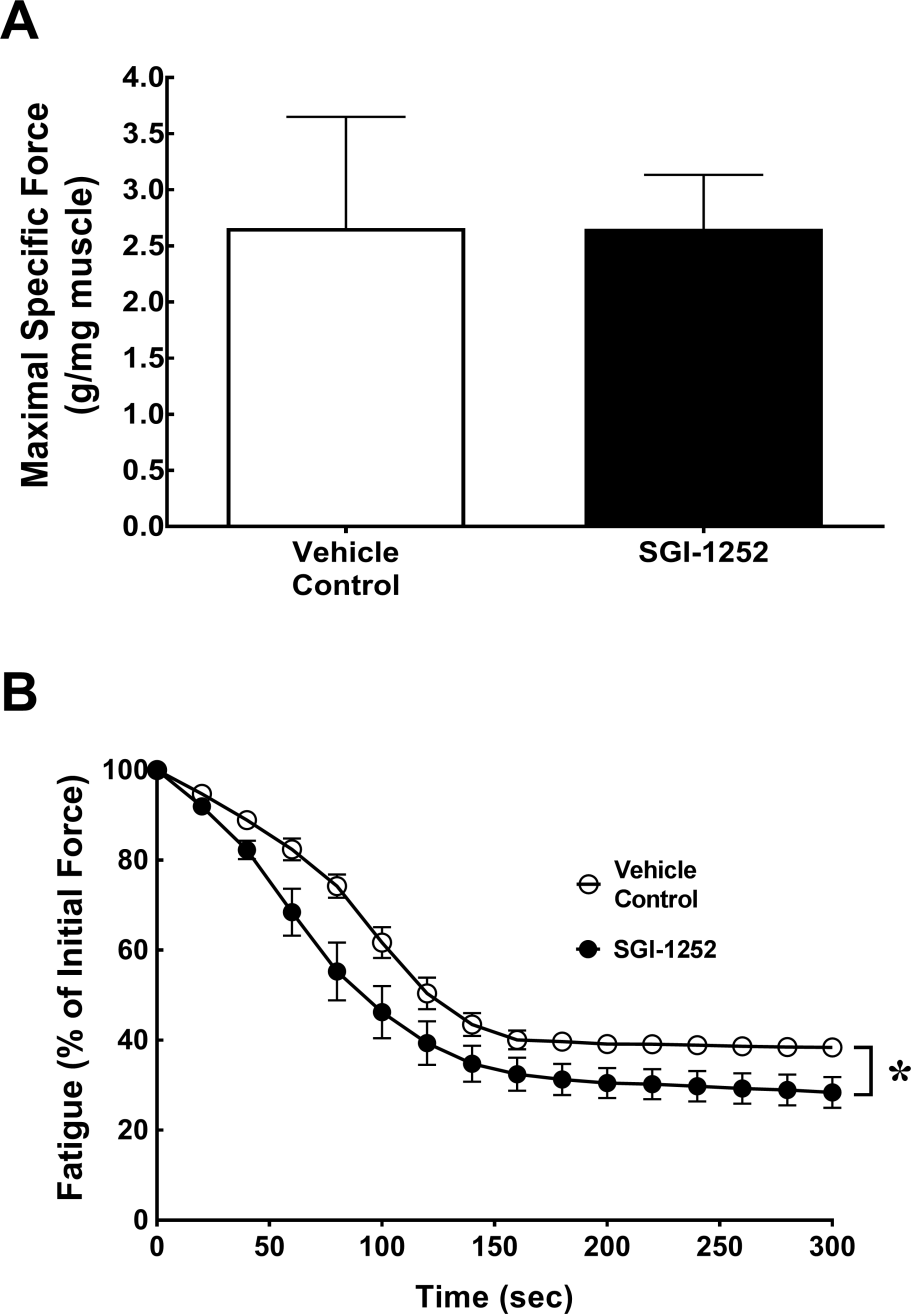
Functional characteristics. (A) Maximal specific force and (B) fatigue were assessed to determine functional characteristics of the gastrocnemius-plantaris-soleus complex. Data are presented as means ± S.D. * Significance of p < 0.05.

Muscle function and fatigue characteristics of the gastrocnemius-soleus-plantaris complex were assessed at the end of the treatment period via an *in-situ* preparation and can be found in Fig 2. During the 300 seconds of tetanic contractions, SGI-1252 treated and control mice showed a similar maximal specific force (2.66 ± 0.99 vs 2.65 0.48 g/mg muscle; p = 0.99)(Fig 2A) and rate of fatigue (p = 0.81, R^2^ = 0.87) (Fig 2B). However, the extent of total fatigue as measured by the percentage of force remaining at the end of the bout was greater in the SGI-1252 (28.4 ± 7.7%) mice relative to controls (37.9 ± 1.5%) evident by the significant main effect for treatment (p = 0.035)(Fig 2B).

### Whole muscle and myofiber size

**Fig 3:**
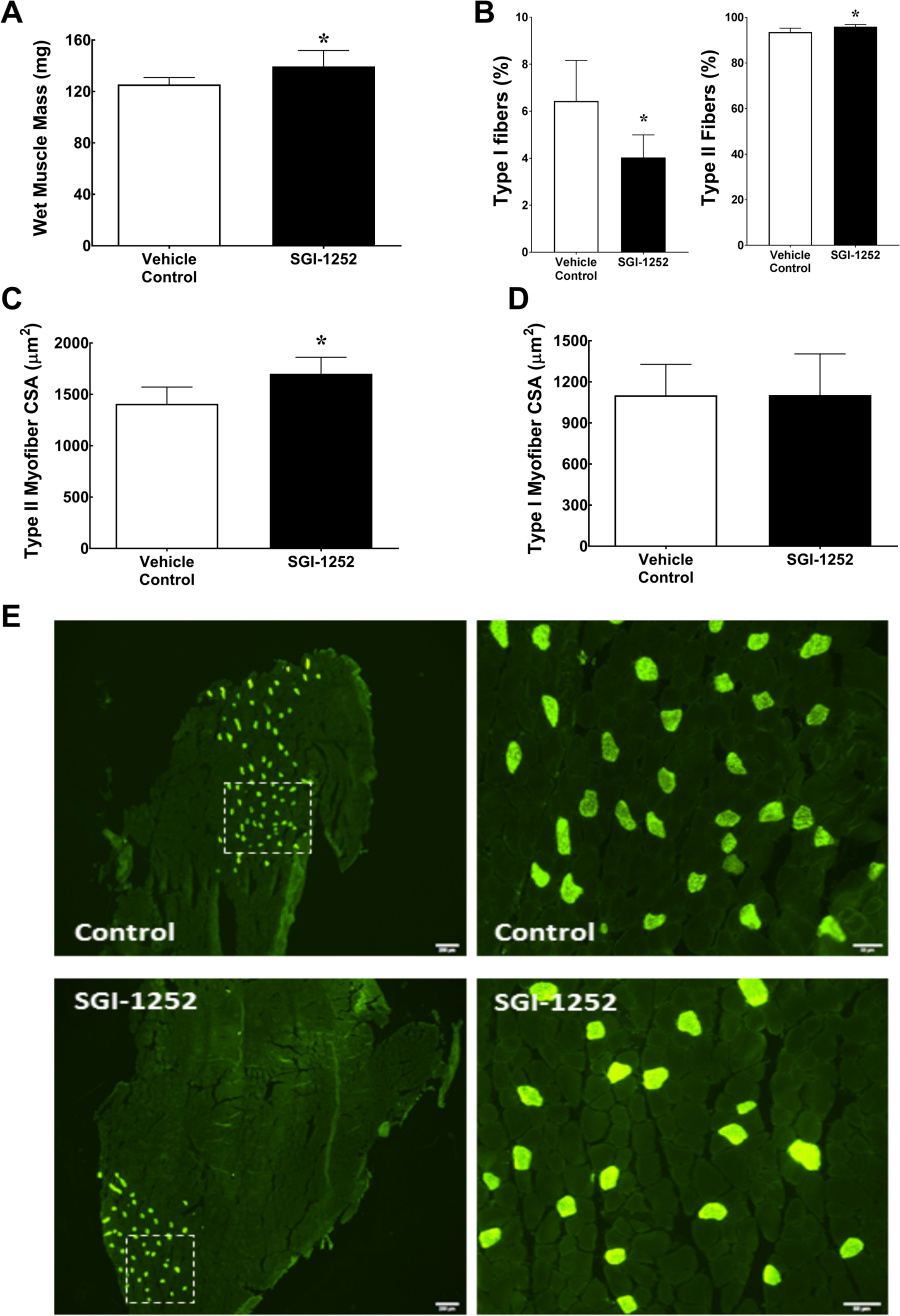
Muscle characteristics. (A) Gastrocnemius wet muscle mass, (B) Proportion of type I and II myofibers, (C) type I and (D) type II myofiber cross-sectional area (mCSA) with (E) Representative images of controls and SGI-1252 gastrocnemius type I (bright green) and type II (dark) myofibers. White square in images denotes the boundaries of the enlarged image. Scale bar = 200 μm and 50 μm respectively. * Significance of p < 0.05.

Despite similar gains in body mass, we found that gastrocnemius wet muscle mass in the SGI-1252 treated mice (139.6 ± 12.34 mg) were 10% greater than control mice (125.45 ± 5.46 mg, p = 0.032)(Fig 3A). Consistent with changes in wet mass, gastrocnemius myofiber cross-sectional area (mCSA) showed a 22% increase in type II myofibers of the SGI-1252 treated mice relative to the controls (VC = 1434.8 ± 225.4 vs SGI-1252 = 1754 ± 138 μm^2^, p = 0.022)(Fig 2C), whereas type I mCSA was similar between groups (VC = 1153.4 ± 239 vs SGI-1252 = 1103 ± 300, p = 0.765)(Fig 3D). The increase in type II mCSA was not the result of an increase in myonuclei, as the proportion of myonuclei per fiber was similar between groups (VC: 1.26 ± 0.61 vs. SGI-1252: 1.08 ± 0.55 myonuclei/fiber). Additionally, the total number of type I myofibers was significantly reduced in the gastrocnemius of the SGI-1252 treated group relative to controls (VC = 69.2 ± 13.4 vs SGI-1252 = 42.8 ± 7.5, p = 0.004) such that the proportion of type I (VC: 6.43 ± 1.73 vs SGI-1252: 4.03 ± 0.95, p = 0.043) and type II (VC: 93.56 ± 1.73 vs SGI-1252: 95.96 ± 0.95, p = 0.043) fibers were different between groups (Fig 3B and 3E).

### STAT3 phosphorylation and satellite cell content

**Fig 4:**
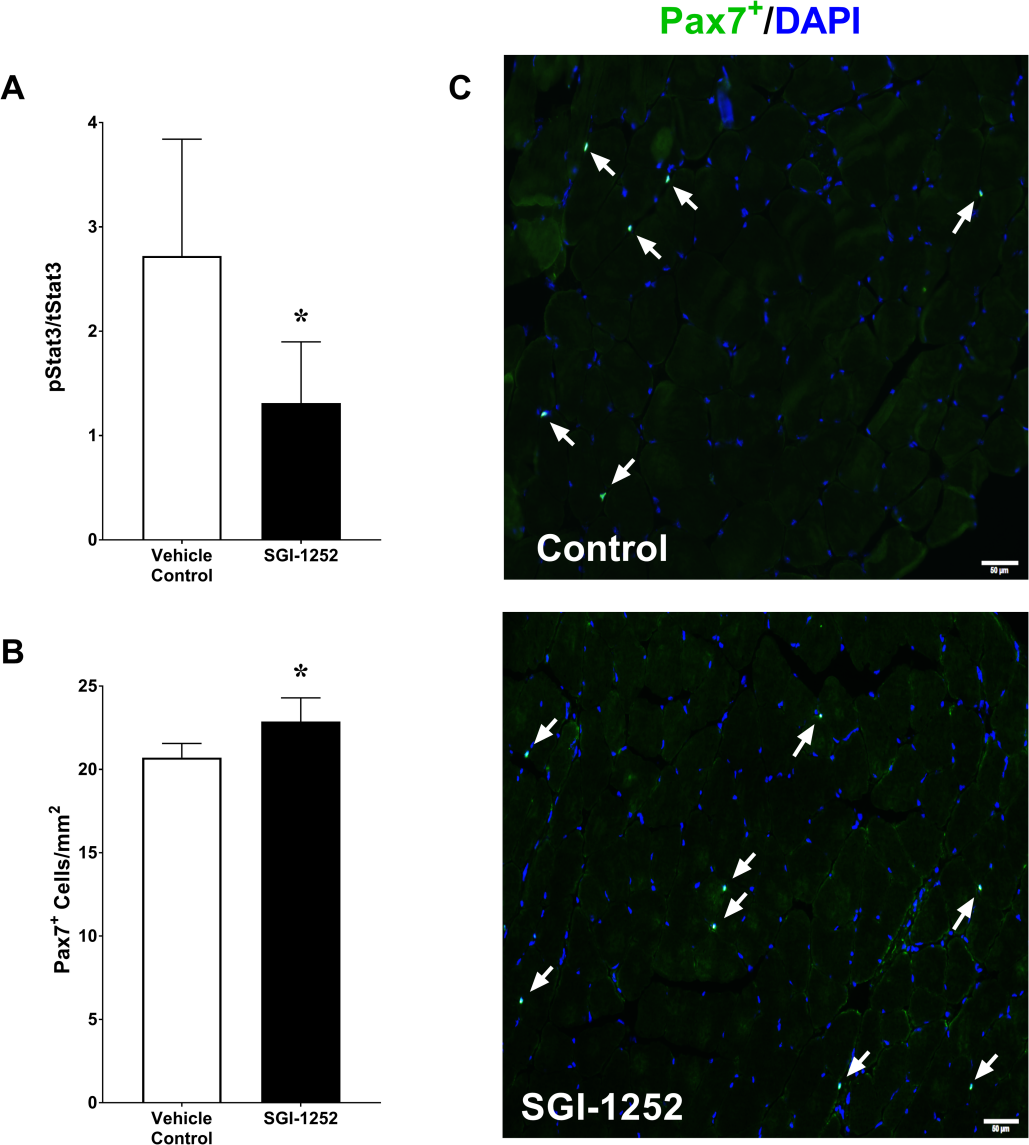
STAT3 and satellite cell concentrations. (A) Phosphorylated-STAT3 displayed as a percentage of the total STAT3 concentration. (B) Histological quantification of Pax7^+^ satellite cells per volume of tissue with a (C) representative image from control and SGI-1252 treated mice. White arrows indicate the location of Pax7^+^ cells. Scale bar = 50 μm. * Significance p < 0.05.

Eight weeks of SGI-1252 treatment led to a 53% reduction in STAT3 phosphorylation (44.5 ± 6.1 MFI) compared to controls (94.79 ± 45.9 MFI, p = 0.022) with no treatment effect on total STAT3 concentrations (VC: 3437 ± 530 vs SGI-1252: 3829 ± 1293 MFI, p = 0.277) (Fig 4A). The proportion of phosphorylated STAT3 to total STAT3 was significantly lower in the SGI-1252 treated animals (VC: 2.72 ± 1.1 vs SGI-1252: 1.31 ± 0.59%, p = 0.014). We further found that inhibition of STAT3 phosphorylation in mice that were treated with SGI-1252 resulted in a significant increase in the number of Pax7^+^ satellite cells relative to controls (VC = 2589.2 ± 105.5 vs SGI-1252 = 2859.4 ± 177.5 SC/mm^3^, p = 0.015)(Fig 4B and 4C).

### Proliferation of human primary myoblasts

**Fig 5:**
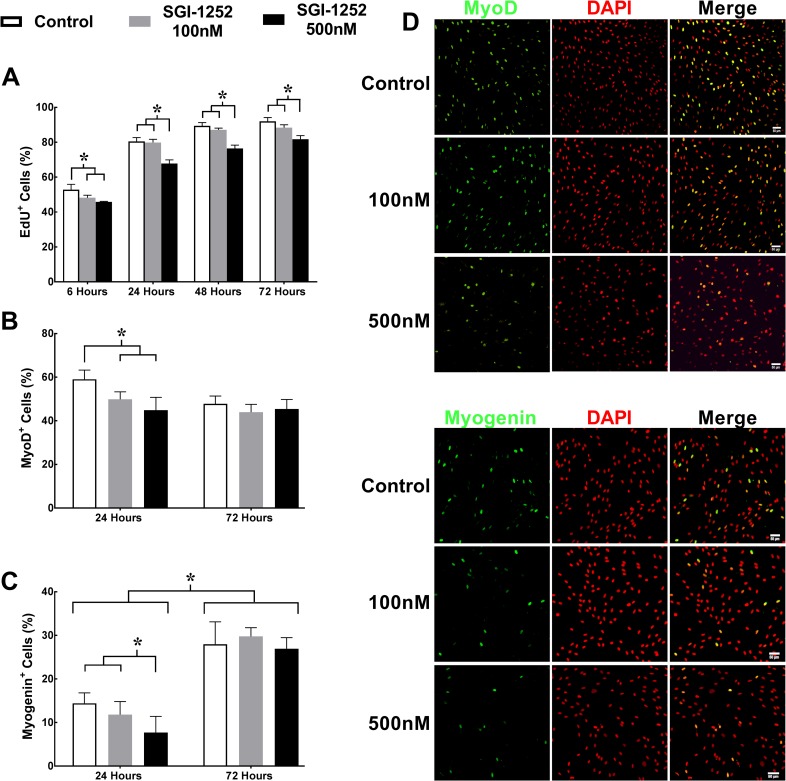
Proliferation and expression of myogenic regulatory factors MyoD and myogenin. (A) Proliferation was assessed using 5-ethynil-2'-deoxyuridine (EdU^+^) following 6, 24, 48, and 72 hours in a growth medium. Myogenic regulatory factors (B) MyoD^+^ and (C) Myogenin^+^ nuclei were assessed following 24 and 72 hours in a differentiation medium. Representative images of (D) MyoD^+^ and (E) Myogenin^+^ nuclei at 24 hours. Scale bar = 50μm. * Significance p < 0.05.

To better understand the mechanisms whereby SGI-1252 treatment affects the satellite cell pool, we performed a series of *in vitro* experiments using isolated human primary myoblast (HPM) cultures. JAK/STAT signaling has been shown previously to regulate the production of myogenic regulatory factors [18] and influence myoblast proliferation [22]. To determine the effect of SGI-1252 on proliferation, we incubated HPM’s in either 100nM SGI-1252, 500nM SGI-1252 or a vehicle control solution, and the percentage of EdU^+^ cells were assessed at 6, 24, 48 and 72 hours (Fig 5). As expected, EdU^+^ nuclei increased over time for all three treatment groups. However, EdU^+^ nuclei in the 100nM and 500nM treatment groups were significantly reduced relative to the control group at the 6-hour time point. At the later time points, the 500nM treated cells remained significantly lower relative to the 100nM and vehicle treated control groups (p<0.001)(Fig 5A). While it appears that proliferation of myoblasts occurred normally in the SGI-1252 treated group, our data do not rule out that SGI-1252 treatment caused increased cell death rates. Specific experiments to test this possibility will need to address this question in the future.

### Myogenic regulatory factors in human primary myoblasts

To assess how SGI-1252 affects myogenic commitment of HPMs, we measured the expression of the myogenic regulatory factors MyoD and Myogenin following 24 and 72 hours of incubation in a differentiation medium (Fig 5B–5E). MyoD^+^ and Myogenin^+^ nuclei, expressed as a percentage of total nuclei, were significantly reduced at 24 hours for the 500nM (44.8 ± 5.9% and 7.8 ± 3.7%) SGI-1252 treated cells, respectively compared to vehicle controls (59 ± 4.3% and 14.4 ± 2.4%), while the 100nM SGI-1252 treated cells were lower in only MyoD^+^ expression (49.8 ± 3.5% and 11.8 ± 3%). After 72 hours in differentiation medium there were no differences between treatment conditions, likely due to degradation of the drug over that time period. However, Myogenin^+^ nuclei had increased significantly from the 24-hour time point for all groups.

### Differentiation of human primary myoblasts

**Fig 6:**
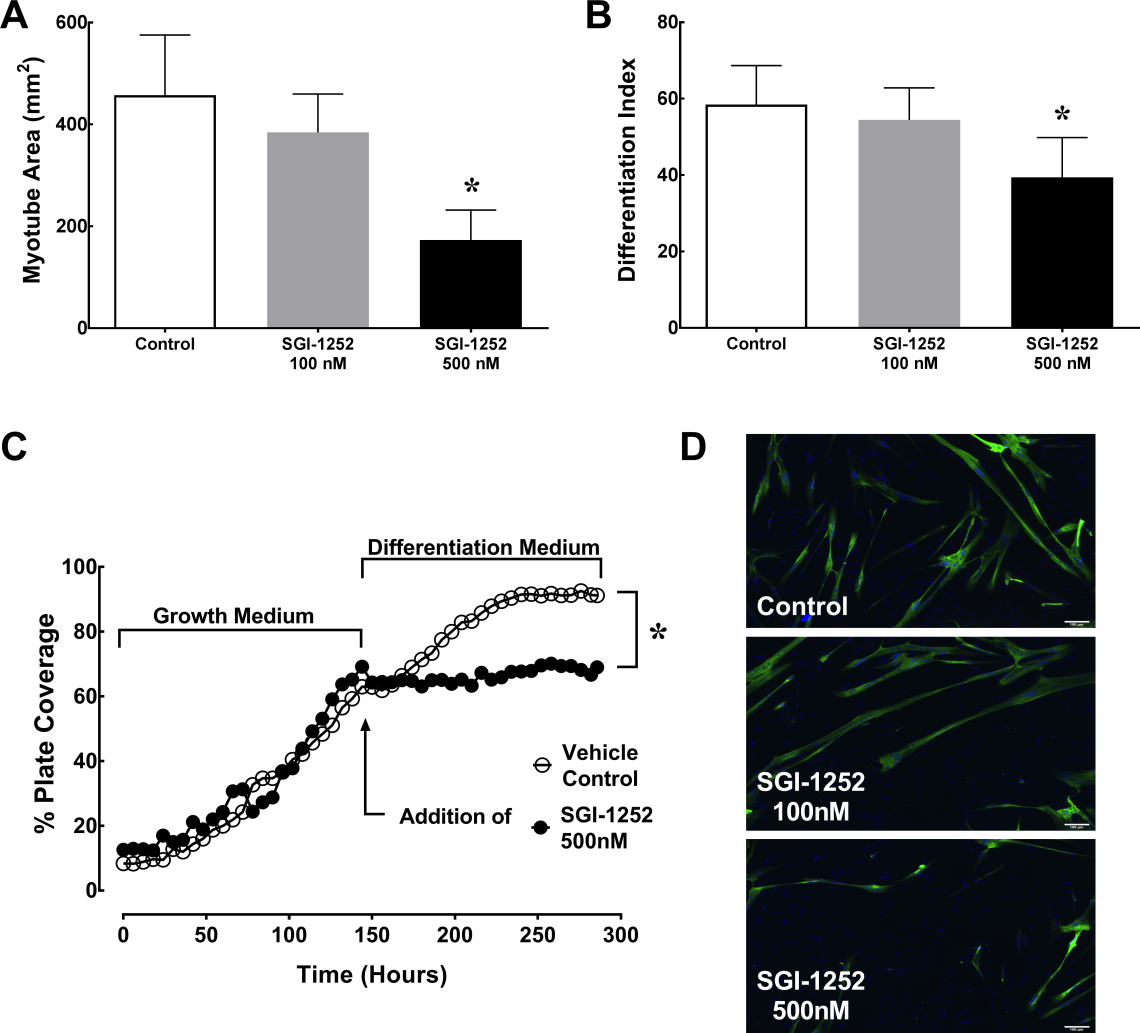
Satellite cell differentiation. (A) Total myotube area and (B) the differentiation index (MyHC^+^ Nuclei / Total Nuclei) were measured to assess myoblast differentiation following 6 days of treatment. (C) Time lapse images measuring plate coverage percentage in a growth and differentiation medium for 12 days with (D) representative images of myosin heavy chain (MyHC^+^) expression (Green), nuclei (Blue) and myotube formation. Scale bar = 100 μm * Significance p < 0.05.

The extent of differentiation was determined by assessing myotube area via myosin heavy chain (MyHC) expression following six days in differentiation medium (Fig 6). Myotube area was significantly lower in the 500nM (163 ± 53 mm^2^) treated group compared to both the 100nM (386 ± 79 mm^2^) and vehicle control (453 ± 124 mm^2^, p = 0.0051) treatment groups (Fig 6A). To verify that the reduction in myotube area was not the result of reduced myoblast proliferation in SGI-1252 treated mice, we calculated the differentiation index (MyHC^+^ nuclei / total nuclei). We measured a significant decline in the 500nM SGI-1252 treatment group (39.4 ± 10.4%) compared to the 100nM SGI-1252 (54.4 ± 8.4%) and vehicle treated control groups (58.5 ± 10.2%, p = 0.039)(Fig 6B). Additionally, time lapse images were taken during a 12-day cell culture experiment to compare differentiation in the 500nM SGI-1252 treatment vs. vehicle control treatment. Percentage of plate coverage was used to assess myogenic progression, which was markedly reduced in the 500nM SGI-1252 treated cells compared to the vehicle control (Fig 6C and S1A and S1B Video). Taken together, these data indicate that SGI-1252 reduces myogenic commitment and differentiation.

## Discussion

The dysfunctional response observed in satellite cell quiescence and self-renewal characteristics have been associated with an elevated immune response to increased JAK/STAT signaling in muscle wasting conditions [24]. Therefore the primary aim of this preclinical study was to determine the feasibility of using the novel small molecule SGI-1252, a known JAK inhibitor [29], to improve skeletal muscle satellite cell function. Additionally, isolated HPM’s were used to identify the mechanism by which SGI-1252 may function to increase *in vivo* satellite cell content. We observed that animals steadily gained weight over the 8-week treatment period and displayed no physical abnormalities or adverse events compared to the control group. As hypothesized, we found that 8 weeks of SGI-1252 treatment significantly reduced skeletal muscle STAT3 signaling activity and increased muscle satellite cell content in the gastrocnemius muscle. Interestingly, we found that 8 weeks of SGI-1252 treatment also increased gastrocnemius wet weight and type II mCSA, and increased fatigue during a 300 second tetanic contraction bout. Using an *in vitro* model of myogenesis we found that SGI-1252 attenuated myogenic commitment and decreased differentiation of HPM’s. This study supports recent findings that indicate STAT3 signaling as a key regulator of satellite cell function, and provides compelling pre-clinical muscle data on a novel, orally bioavailable JAK inhibitor.

STAT3 signaling is primarily activated by the binding of extracellular interleukins (Interleukin-6 and leukemia inhibitory factor) and growth factors (basic fibroblast growth factor and platelet-derived growth factor), secreted during inflammation and exercise, to regulate metabolic responses and stimulate muscle repair [14]. Chronically elevated STAT3 activity can lead to downstream activation of myostatin [39] and the ubiquitin proteasome system, as observed in cancer cachexia models [40], causing protein degradation and loss of muscle mass. In the present study we found that oral administration of SGI-1252 significantly reduced STAT3 activity in mouse gastrocnemius muscle. The decline in STAT3 phosphorylation via SGI-1252 was measured in healthy mice, and indicate the promising potential that the molecule may reduce STAT3 activity under pathologic conditions characterized by elevated STAT3 signaling.

In conjunction with attenuated STAT3 activity, we found a significant expansion of Pax7^+^ satellite cells in the SGI-1252 treated mice. Tierney et al. [18] also showed satellite cell expansion in the muscle of dystrophic and aged mice that were acutely treated with a STAT3 inhibitor, which was validated *in vitro* using both human and mice myoblasts [17]. Interestingly, they showed that STAT3 inhibition not only led to satellite cell expansion, but accelerated regeneration in aged mice and larger regenerating fibers were detected in dystrophic mice. These data provide strong evidence that an expansion to the satellite cell pool improves muscle regenerative capacity. It has also been suggested that satellite cells contribute to muscle fibers during the regular maintenance process of aging, even in the absence of injury [33]. Taken together, intermittent use of SGI-1252 may improve skeletal muscle function by expanding the satellite cell pool in conditions characterized by elevated JAK/STAT signaling and satellite cell dysfunction.

To further explore how JAK/STAT inhibition via SGI-1252 treatment could increase the satellite cell pool in healthy mice, we performed *in vitro* experiments using HPM cultures. Studies show that STAT3 activity impedes symmetrical expansion of the satellite cell population [17]. Conversely, STAT3 inhibition increases self-renewing potential by attenuating transcription factors (MyoD and Myogenin) that promote satellite cell differentiation [41]. Consistent with these studies, we measured a significant decrease in the expression of MyoD and Myogenin in SGI-1252 treated myoblasts, which was further supported by an attenuation in myoblast differentiation (myotube area, differentiation index). Typically, a robust response in satellite cell activity is beneficial to promote efficient muscle regeneration following exercise and injury, yet chronic activation can be detrimental. This phenomenon is observed during the functional decline of aged, sarcopenic muscle [10]. Along these lines, it has been suggested that STAT3 inhibition provides a means whereby chronically active satellite cells can return to quiescence and retain their functional capacity [18]. Correspondingly, we also measured an attenuated proliferative response in SGI-1252 treated myoblasts that is supported by *in vivo* studies [42], yet not consistent with some *in vitro* findings. We believe that this discrepancy may be due to differences in the health and species from which the cells were collected. As the myoblasts used in our study came from a young, healthy man, other studies have measured proliferation in aged or diseased rodents. Nonetheless, we suspect that the reduced proliferative response observed in our study is likely attributed to an upregulation of quiescent factors in *de novo* daughter cells to limit cell cycle activity. Thus, activated satellite cells in the SGI-1252 treated mice can more readily return to quiescence and repopulate the niche. Furthermore, by limiting activation, characteristics that increase their regenerative and functional capacity are preserved.

An interesting and somewhat unexpected finding was that SGI-1252 treatment resulted in a significant increase in CSA of type II myofibers, and that overall, SGI-1252 treatment resulted in a larger fraction of type II fibers and a reduced proportion of type I fibers. It is likely that the shift in fiber type led to the increased overall muscle fatigability in the SGI-1252 mice during the 300 second tetanic contraction bout. The mechanism driving an increase in mCSA cannot be ascertained with our data, but it is unlikely that increased satellite cell content in the treated mice led to increased mCSA, as we show here that JAK/STAT inhibition expands the satellite cell pool by diminishing myogenic differentiation. However, activation of the JAK/STAT signaling pathway has been implicated in a variety of atrophy-inducing stimuli including mechanical ventilation [43] and cancer cachexia [44]. For example, in cancer cachexia, activated STAT3 has been shown to initiate skeletal muscle protein loss via the stimulation of caspases, myostatin and the ubiquitin-proteasome system [40]. Thus, it is possible that chronic STAT3 inhibition in healthy mice could have inhibited the breakdown of protein, resulting in an increase in type II myofiber size over the 8-week treatment period, especially given the preferential activity of STAT3 in type II fibers [45]. Interestingly, the larger myofiber CSA did not translate to an increase in maximal specific force production in the SGI-1252 treated mice, indicating that the quality of the muscle was not altered by the drug. A similar phenomenon has been observed in myostatin deficient mice, which display a robust increase in fiber cross-sectional area with little to no change in maximal isometric specific force [46]. Additionally, as wet mass and type II mCSA increased in the SGI-1252 treated mice despite a similar rise in body mass, we speculate that inhibition of STAT3 may have reduced fat mass in the SGI-1252 treated mice, as studies have shown an association between STAT3 signaling, inflammation and obesity [47], yet we are unable to make this conclusion due to the lack of data. Furthermore, though not statistically different, there was a trend for increased body mass over time that partially supports the increase in muscle mass. Furthermore, as previous studies have demonstrated that the use of STAT3 inhibitor was accompanied by improved muscle function [17], it is important to note that discrepancies likely exist due to differences in the health of the animals at the time when the test were administered. Specifically, functional tests from our study were performed on healthy mice following an 8-week treatment period, whereas previous studies assessed functional capacity following cardiotoxin induced muscle injury as a means of evaluating the regenerative capacity of the muscle. We speculate that an expansion in the satellite cell pool, as observed in our SGI-1252 treated mice, would likewise result in an increase in functional capacity following injury. Nonetheless, based on the current data, we suggest that JAK/STAT inhibition via SGI-1252 may not be an ideal therapeutic intervention to treat muscle functional declines, though studies under pathological conditions would be needed to truly ascertain its functional therapeutic value.

It is also important to note the limitation that, in this study, we only assessed the effect of SGI-1252 on muscles of the plantar flexor group, particularly the gastrocnemius. This is potentially important, as Keefe et al. demonstrated that both satellite cell content, as well as their contribution (i.e., fusion) to healthy, uninjured muscle varies between muscles [30]. However, as it relates to the results of our study, contrary to the other lower limb muscles (e.g. TA, EDL, plantaris), Keefe et al. showed that satellite cell content of the gastrocnemius decreases 16–25% by 20 months of age. They further showed that contribution of satellite cells to healthy muscle increased between 12 and 20 months of age only in the gastrocnemius, and not the other lower limb muscles. These findings underscore the potential importance of healthy satellite cell function to gastrocnemius well-being over the lifespan. Nevertheless, future studies should be directed at investigating the effect of SGI-1252 on other muscles and muscle groups.

## Conclusion

SGI-1252 was effective in attenuating STAT3 signaling and promoting satellite cell expansion with no loss in maximal force production or increased rate of fatigue during the contraction bout. We further show an attenuated myoblast differentiation response with SGI-1252 treatment, thus providing a potential mechanism for the expansion of the satellite cell pool *in vivo*. As recent studies have suggested, STAT3 inhibition is a potential target in combating the negative effects associated with satellite cell dysfunction and muscle wasting conditions by restoring characteristics of self-renewal and quiescence to the active satellite cell pool. Our findings suggest that SGI-1252 may provide beneficial health effects in the treatment of several health conditions characterized by increased STAT3 activity, and that further research is warranted in these populations.

## Supporting information

S1 Video mp4 vehicle control 12 day cell culture experiment. (MP4)

S2 Video mp4 500nM SGI-1252 treated 12 day cell culture experiment. (MP4)
